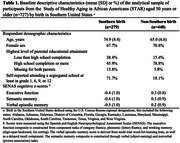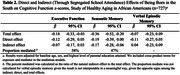# Evaluating segregated school attendance as a mediator of geographic inequities in late‐life cognitive function: evidence from the Study of Healthy Aging in African Americans (STAR)

**DOI:** 10.1002/alz.091902

**Published:** 2025-01-09

**Authors:** Sirena Gutierrez, Rachel A. Whitmer, Yi Lor, Kristen M. George, Lisa L. Barnes, Elizabeth Rose Mayeda, Isabel Elaine Allen, M. Maria Glymour, Jacqueline M Torres, Paola Gilsanz

**Affiliations:** ^1^ University of California San Francisco, San Francisco, CA USA; ^2^ University of California, Davis, Davis, CA USA; ^3^ Rush University Medical Center, Chicago, IL USA; ^4^ UCLA Fielding School of Public Health, University of California, Los Angeles, CA USA; ^5^ Boston University School of Public Health, Boston, MA USA; ^6^ Kaiser Permanente Northern California Division of Research, Oakland, CA USA

## Abstract

**Background:**

Being born in the Southern US is associated with poorer cognitive health and related cardiovascular outcomes in late life, especially among Black Americans. Geographic disparities in brain aging may be mediated by school segregation; school segregation has been disproportionately high in the South given historical and contemporary policies and practices.

**Method:**

This study included 727 Black adults ages 50+ (mean 68.8 (8.8±SD) years) living in Northern California from the Study of Healthy Aging in African Americans (2018). We employed linear regression models to estimate associations between Southern US birth and domain‐specific cognitive function z‐scores, adjusting for early‐life covariates. Utilizing mediation analysis, we decomposed the total effect of Southern birth into natural direct effects and natural indirect effects via self‐reported ever attending a segregated school at grade 1, 6, 9, or 12.

**Result:**

The 38% of participants born in the South averaged older age (74.9 vs. 65.0 years), had less educated parents (38.4% vs. 15.4% with less than high school), and were more likely to have attended segregated schools (71.7% vs. 18.1%) compared to those born in other regions. Southern birth was associated with lower late‐life executive function (β[95% CI] = ‐0.18 [‐0.33, ‐0.03]) and semantic memory (‐0.36 [‐0.52, ‐0.20]); estimates were imprecise for verbal episodic memory (‐0.07 [‐0.23, 0.09]). Under the assumptions for mediation analyses, the estimated *direct* effect of Southern birth, not mediated by attending a segregated school, was ‐0.12 [‐0.29, 0.05] for executive function, ‐0.19 [‐0.39, 0.00] for semantic memory, ‐0.11 [‐0.27, 0.05] for verbal episodic memory. Attending a segregated school mediated 38% and 47% of the total effect of Southern birth on executive function (indirect effect: ‐0.07 [‐0.17, 0.03]) and semantic memory function (indirect effect: ‐0.17 [‐0.28, ‐0.06]).

**Conclusion:**

School segregation may be an important mechanism underlying geographic inequities in late‐life health in the US. Interventions to reduce persistent *de facto* school segregation may help ameliorate these inequities. Future research should consider other mechanisms related to the schooling environment (e.g. resources, quality) that may be additional targets of intervention.